# Effectiveness, Safety, and Patient Satisfaction of Liraglutide in Type 2 Diabetic Patients

**DOI:** 10.7759/cureus.9937

**Published:** 2020-08-22

**Authors:** Rafia Zameer, Matiullah Kamin, Umar Raja, Muhammad Umar Wahab, Osama Ishtiaq, Kashif Raashid, Naveed Ahmed, Asim.ur. Rehman

**Affiliations:** 1 Pharmacy, Quaid-I-Azam University, Islamabad, PAK; 2 Endocrinology, Shifa International Hospital, Islamabad, PAK; 3 Endocrinology, Umar Diabetes and Footcare Clinic, Islamabad, PAK; 4 Diabetology, Umar Diabetes Foundation, Islamabad, PAK

**Keywords:** liraglutide, hemoglobin a1c, retrospective study, type 2 diabetes mellitus

## Abstract

Background and Objectives

Liraglutide, an analog of human glucagon-like peptide 1 (GLP-1), has been approved for the treatment of type 2 diabetes mellitus in Pakistan since 2016. It is a GLP-1 receptor agonist that has shown promising results in terms of not only glycemic control but also weight loss. Our study aimed to provide evidence regarding the safety and effectiveness of liraglutide in Pakistan and to look at the adherence rate and treatment satisfaction of patients using liraglutide.

Methods

This is an observational retrospective study that recruited patients who were treated with liraglutide. Data were collected at the first visit and follow-up. Morisky Green Levine Adherence Scale and Treatment Satisfaction Questionnaire for Medication (TSQM-9) were used for the determination of adherence and satisfaction with the treatment.

Results

A total of 70 patients were recruited in the study, The mean difference in weight, body mass index, glycated hemoglobin (HbA1C), systolic blood pressure, and diastolic blood pressure from baseline to follow-up was -5.36 kg, -2.14 kg/m^2^, -1.76%, -12.38 mmHg, and 5.55 mmHg, respectively. Nausea was the main side effect reported. TSQM-9 scores were compared from baseline, and it was found that patients are satisfied with the treatment and its effectiveness.

Conclusions

Our study has demonstrated the effectiveness of liraglutide as a monotherapy or combination therapy in the Pakistani population. Liraglutide led to reduction in HbA1C and weight. This is associated with high treatment satisfaction rate and adherence rate. Thus, liraglutide remains an effective though expensive treatment option in a country like Pakistan.

## Introduction

Type 2 diabetes mellitus (T2DM) is a complex metabolic disorder with multiple pathophysiological mechanisms [1]. With increase understanding of the pathophysiological mechanisms behind T2DM, there have been several new treatment options over the last decade or so. One such treatment option is glucagon-like peptide 1 (GLP-1) receptor agonists that have shown promising results in terms of not only glycemic control but also weight loss [2].

Injectable GLP-1 receptor agonist mimics endogenous GLP-1 by way of stimulating pancreatic insulin secretion as well as suppressing glucagon production [2]. Regardless of its function in glucose homeostasis, the GLP-1 receptors are widely distributed throughout the body, including the heart, brain, gastrointestinal tract, and pancreas. GLP-1 may exert its outcomes through both receptor-dependent and receptor-independent mechanisms and through the actions of both the intact peptide and its metabolites [3]. GLP-1 receptor agonists have shown cardiovascular benefits as well as a reduction in albuminuria independent of glycemic control, causing a new dimension within the control of T2DM [4].

Liraglutide, an analog of human GLP-1, has been approved for the treatment of T2DM in Pakistan since 2016 [5]. Although there are real-world data about the safety and effectiveness of liraglutide worldwide, there is a dearth of such data in South Asia. So far, there have been small-scale studies in India and Pakistan looking at only the safety and effectiveness of liraglutide [5,6]. Our study aimed not only to provide real-world evidence regarding the safety and effectiveness of liraglutide in the Pakistani population but also to look at adherence rate and treatment satisfaction of patients using liraglutide.

## Materials and methods

This is an observational retrospective study that recruited patients from endocrinology clinics of Shifa International Hospital Islamabad and Umar Diabetes and Foot Care Centre, Islamabad, Pakistan, from September 2018 to June 2019. The study was conducted after approval by the respective Ethical Review Committees. All patients with T2DM above 18 years with glycated hemoglobin (HbA1c) ≥ 7% and body mass index (BMI) ≥ 25 kg/m^2^ were prescribed liraglutide 0.6 mg subcutaneously once a day irrespective of their meal at the same time initially for the first five days, and from the sixth day they were switched to a dosage of 1.2 mg with further up-titration to 1.8 mg based on tolerance. Other prescribed drugs were advised to be taken accordingly. Patients were excluded if they had type 1 diabetes and a glomerular filtration rate (GFR) < 45 mL / min / 1.73 m^2^.

At first visit, baseline data were collected using a pre-designed data collection form, which included age, weight, BMI, blood pressure, and HbA1c. Morisky Green Levine (MGL) Adherence Scale and Treatment Satisfaction Questionnaire for Medication (TSQM-9) were used for the determination of adherence and satisfaction with the treatment [7,8]. Analysis was based on efficacy endpoints that were reduction in HbA1c, BMI, and fasting glucose levels from baseline values.

Statistical analyses were performed using IBM SPSS Statistics for Windows, Version 20.0 (IBM Corp., Armonk, NY, USA). Categorical variables were recorded as frequencies and percentages for each category and continuous variables were recorded as means and standard deviations. A paired t-test was applied for pre-post changes in clinical outcomes and treatment satisfaction, whereas analysis of variance (ANOVA) was applied to determine the differences between various diabetics groups.

## Results

A total of 70 patients were recruited in the study, out of which 52.9% were males and 41.1% were females with a mean diabetes duration of 8.53 ± 6.44 years. Baseline characteristics are given in Table 1. Around 85% of the patients were taking 1.2 mg of liraglutide once a day. In addition to liraglutide, patients were also reported to take metformin, sodium-glucose co-transporter-2 (SGLT2) inhibitors, sulfonylureas, and insulin. Most of the patients were moderately controlling their diet but were not exercising.

**Table 1:** Descriptive analysis of patients on liraglutide at baseline SD, standard deviation; CVD, cardiovascular disease; BMI, body mass index; SGLT2, sodium-glucose co-transporter-2; HbA1C, glycated hemoglobin; SBP, systolic blood pressure; DBP, diastolic blood pressure

S. No.	Variables	n (%)
1	Age (Years), Mean ± SD	49.35 ± 10.91
2	Gender	
	Male	37 (52.9)
	Female	33 (47.1)
3	Duration of Diabetes (Years), Mean ± SD	8.53 ± 6.44
4	Comorbidity	
	No Comorbidity	28 (40)
	Hypertension	24 (34.3)
	CVD	1 (1.4)
	Cholesterol	6 (8.6)
	Hypertension + Cholesterol	5 (7.1)
	Hypertension + Cholesterol + CVD	3 (4.3)
	Others	3 (4.3)
5	Weight (kg), Mean + SD	93.70 ± 3.92
6	BMI (kg/m^2^), Mean ± SD	33.74 ± 5.89
7	Dosage of Liraglutide	
	0.8 mg	1 (1.4)
	1.2 mg	60 (85.7)
	1.8 mg	9 (12.9)
8	Medication History	
	Liraglutide	18 (25.7)
	Liraglutide + SGLT2 Inhibitors	17 (25.7)
	Liraglutide + Sulfonylureas	7 (10.0)
	Liraglutide + Metformin	2 (2.9)
	Liraglutide + Metformin + SGLT2 inhibitors	5 (7.1)
	Liraglutide + Pre-Mix Insulin	9 (12.9)
	Liraglutide + Long-Acting Insulin	18 (25.7)
9	HbA1C (%), Mean ± SD	9.57 ± 2.45
10	SBP (mmHg), Mean ± SD	136.78 ± 17.63
11	DBP (mmHg), Mean ± SD	88.6 ± 10.49
12	Blood Urea (mg/dL), Mean ± SD	29.33 ± 11.15
13	Creatinine (mg/dL), Mean ± SD	0.8 ± 0.22
14	Diet	
	Poor Control	25 (35.7)
	Moderate Control	40 (57.1)
	Strict Control	5 (7.1)
15	Exercise	
	No	42 (60.0)
	Moderate	27 (38.6)
	Strict	1 (1.4)

Table 2 shows the comparison of variables from baseline to follow-up of patients taking liraglutide. The mean difference in weight, BMI, HbA1C, systolic blood pressure, and diastolic blood pressure from baseline to follow-up is -5.36 kg, -2.14 kg/m^2^, -1.76%, -12.38 mmHg, and 5.55 mmHg, respectively. When inquired on follow-up, majority of the patients reported having nausea (48%) as a side effect of liraglutide, and a few reported to experience diarrhea, vomiting, and headache as well.

**Table 2:** Comparison of variables from baseline to follow-up of liraglutide treatment BMI, body mass index; HbA1C; glycated hemoglobin

S. No	Variable	At Baseline (Mean)	At 3-6 Months Follow-up (Mean)	Difference (Mean)
1	Weight (kg)	93.70	88.34	-5.36
2	BMI (kg/m^2^)	33.74	31.6	-2.14
3	Blood Pressure			
	Systolic Blood Pressure (mmHg)	136.78	124.4	-12.38
	Diastolic Blood Pressure (mmHg)	88.6	83.05	-5.55
4	HbA1C (%)	9.57	7.81	-1.76
5	Fasting Plasma Glucose (mg/dL)	213.57	124.4	-89.17
6	Side Effects, n (%)			
	Nausea		34 (48.6)	
	Diarrhea		2 (2.9)	
	Vomiting		2 (2.9)	
	Headache		1 (1.4)	
	Nausea + Diarrhea		1 (1.4)	
	Nausea + Vomiting		5 (7.1)	
	Nausea + Headache		1 (1.4)	

TSQM-9 scores were compared from baseline, and it was found that patients are satisfied with the treatment and its effectiveness (p<0.05), whereas in terms of convenience, there was no significant difference (Table 3).

**Table 3:** PROs: TSQM-9 PROs, patient-reported outcomes; TSQM-9, Treatment Satisfaction Questionnaire for Medication

S. No.	PROs	Baseline	Follow-up
1	Domain 1: Effectiveness	50.95	71.34
2	Domain 2: Convenience	69.06	71.34
3	Domain 3: Global Satisfaction	47.95	69.38

**Figure 1:**
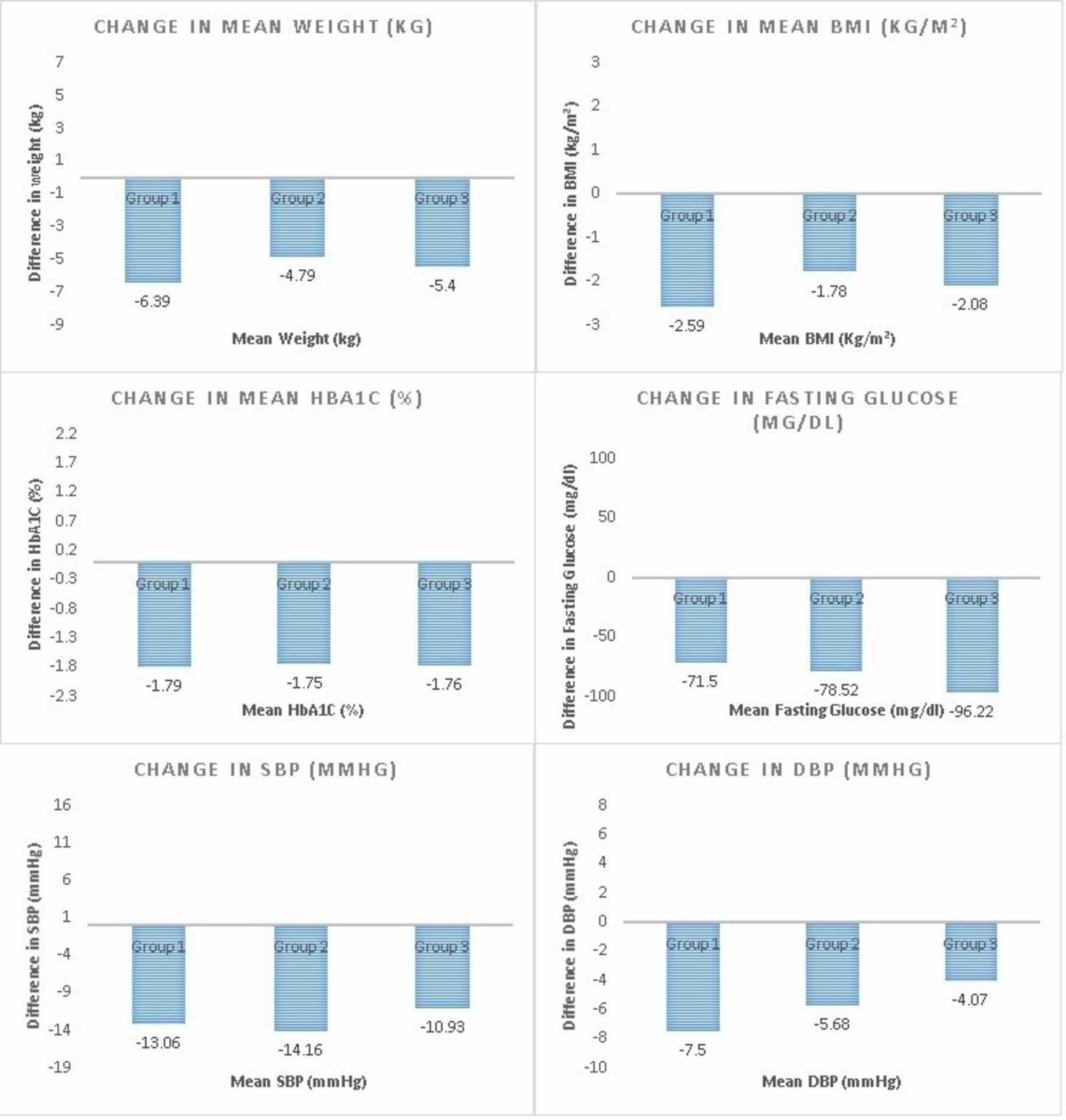
Mean changes in baseline characteristics in different groups SBP, systolic blood pressure; DBP, diastolic blood pressure

Of the patients, 18 were prescribed liraglutide only, 25 liraglutide and oral anti-diabetics (SGLT-2 inhibitors, metformin, sulfonylurea), and 27 liraglutide and insulin. These three groups were assessed individually (Table 4), showing significant results, and collectively no significant changes were observed in terms of glycemic levels (p > 0.05) but changes in weight were identified in the insulin group as well (Table 5). Figure 1 shows the difference in baseline values comparing all three therapy groups.

**Table 4:** Comparison of Liraglutide with OADs and Insulin BMI, body mass index; HbA1C, glycated hemoglobin; OADs, oral anti-diabetics

S. No	.Variables	Pre-treatment (Mean)	Post-treatment (Mean)	Difference	p-Value
	Group 1: Liraglutide as Monotherapy (n=18)				
1	Weight	91.77	85.38	6.39	<0.001
2	BMI	32.85	30.26	2.59	<0.001
3	HbA1C	8.58	6.79	1.79	<0.001
4	Fasting Glucose Levels	189.72	118.22	71.5	<0.001
5	Systolic Blood Pressure	130.83	117.77	13.06	0.001
6	Diastolic Blood Pressure	88.61	81.11	7.50	<0.001
	Group 2: Liraglutide with OADs (n=25)				
1	Weight	93.88	89.09	4.79	<0.001
2	BMI	33.36	31.58	1.78	<0.001
3	HbA1C	9.73	7.98	1.75	<0.001
4	Fasting Glucose Levels	202.92	124.40	78.52	<0.001
5	Systolic Blood Pressure	140.56	126.40	14.16	<0.001
6	Diastolic Blood Pressure	89.52	83.84	5.68	0.008
	Group 3: Liraglutide with Insulin (n=27)				
1	Weight	95.00	89.60	5.4	<0.001
2	BMI	34.78	32.70	2.08	<0.001
3	HbA1C	10.08	8.32	1.76	<0.001
4	Fasting Glucose Levels	239.25	143.03	96.22	<0.001
5	Systolic Blood Pressure	137.22	126.29	10.93	<0.001
6	Diastolic Blood Pressure	87.77	83.70	4.07	0.068

**Table 5:** Comparison of three liraglutide groups using one-way ANOVA BMI, body mass index; HbA1C, glycated hemoglobin; ANOVA, analysis of variance

S. No.	Variable	Variance	Significance
1	Weight	0.008	0.997
		2.349	
2	BMI	13.523	0.415
		15.188	
3	HbA1C	3.461	0.133
		1.664	
4	Fasting Glucose Levels	3790.982	0.220
		2450.111	
5	Systolic Blood Pressure	70.093	0.702
		197.196	
6	Diastolic Blood Pressure	63.826	0.503
		91.907	

## Discussion

This study shows that liraglutide is beneficial in reducing weight, BMI, HbA1C levels, fasting blood glucose, and blood pressure in Pakistani patients with T2DM. Liraglutide has been shown in LEAD (Liraglutide Effect and Action in Diabetes) trials to improve glycemic control, with HbA1c reduction of up to 0.8-1.6%. We also observed a significant reduction in HbA1C levels similar to the outcome of LEAD trials [9-13]. In Italy, a cohort of 400 patients was prescribed with liraglutide and showed a significant positive determinant for HbA1c reduction after a follow-up of 12 months [14]. Another study reported HbA1C levels decreased from 7.9 ± 0.9% at baseline to 7.0 ± 0.7% after five years of liraglutide treatment [15]. Fasting plasma glucose (FPG) was also reported to have significant reduction. There was evidence of a dose-dependent impact of liraglutide on FPG, which was more markedly decreased with the 1.8 mg dose than with the 1.2 mg dose [16]. Another study showed a significant reduction in patients’ FPG after 28 weeks of liraglutide use [17].

Incretin-based treatments can produce weight loss in contrast to many alternative treatment plans that often lead to weight gain. In our study, liraglutide also showed its efficacy in weight and BMI reduction when used as a monotherapy or in combination with other oral hypoglycemic agents and insulin. The mean weight and BMI reduction at follow-up from baseline in our study were 5.36 kg and 2.14 kg/m2, respectively. As reported in the LEAD-4 trial, a considerable and sustained weight loss with liraglutide monotherapy was stated in contrast with glimepiride. Weight reduction with liraglutide monotherapy happened usually within the first 16 weeks but was then sustained [12]. Weight loss with liraglutide tends to be dose-dependent both in monotherapy and combination regimens. Throughout the LEAD trials, an analysis of change in body weight stratified through BMI (≥30 vs. <30 kg/m2) established that there has been a greater weight reduction in patients with high BMIs [9-13]. Our study shows similar results.

Liraglutide treatment was associated with reduced systolic and diastolic blood pressure. We observed that the mean systolic and diastolic blood pressure reduction at follow-up from baseline in our study was 12.38 mmHg and 5.55 mmHg, respectively. Similar results were observed in our three different therapy groups. These differences in blood pressure were much higher in our study than reported in LEAD studies that showed a reduction of systolic blood pressure between 2.7 and 2.9 mmHg [9-13]. Another randomized controlled trial analyzed the effect of liraglutide on blood pressure and compared it with placebo, showing a reduction in systolic blood pressure by -5.73 mmHg and a neutral effect on diastolic blood pressure by a mean difference of -1.42 mmHg [18]. Interestingly, a real-world evidence study from India showed a systolic blood pressure reduction of 9.7 mmHg, which was similar to that in our study [19]. We propose that this greater reduction in blood pressure shown in our study was due to greater weight loss (5.36 kg) seen in our cohort due to liraglutide.

In addition to glycemic and metabolic benefits, liraglutide is well known for gastrointestinal side effects including nausea, vomiting, and diarrhea [20]. Most (48%) of the patients in our study also reported nausea. Despite this, it was important to note that our study cohort has shown a high adherence rate to liraglutide, with 55% showing high adherence and 41% medium adherence as per the MGL Adherence Scale. Also, the treatment satisfaction rate in our cohort was high with TSQM-9, showing an increase in effectiveness from baseline 50.95 to 71.34 and global satisfaction from 47.95 to 69.38. However, convenience remains unchanged from 69.06 to 71.34. We, therefore, propose that though liraglutide treatment leads to increase treatment satisfaction and hence a high adherence rate, convenience does not improve due to liraglutide given as a subcutaneous injection once daily.

Strengths and Limitations

This is one of the few studies that not only evaluate the safety and effectiveness of liraglutide in the Pakistani population but also look at treatment satisfaction and adherence rates. Use of liraglutide has raised the level of treatment satisfaction through better glycemic control and had efficacious treatment adherence. However, the study possesses some limitations being an observational cohort study. Firstly, as data were collected retrospectively, the interval between HbA1c measurements was not constant. Secondly, patient records did not mention whether insulin doses were reduced after initiating liraglutide. Thirdly, as this study analyzed patients treated with liraglutide for a short period, it was not possible to predict therapeutic persistence. Fourthly, although commending BMI and HbA1C levels were reported at follow-up, several confounders such as diet and physical activity could not be monitored in a retrospective study. Lastly, due to the shorter duration of this study, we could not evaluate the cardiovascular and renal benefit of liraglutide in our population. Prospective studies are required to further evaluate the safety, effectiveness, and patient satisfaction of liraglutide, comparing multiple useful consequences of liraglutide in patients with cardiovascular risks and baseline albuminuria and thus evaluating the new cardio defensive and renal protecting role of this drug in South Asian population.

## Conclusions

Our study has demonstrated the effectiveness of liraglutide as a monotherapy or combination therapy in the Pakistani population. Liraglutide provided HbA1c reductions in addition to rapid and sustained reductions in FPG, weight loss, and systolic blood pressure. This is associated with high treatment satisfaction rate and adherence rate. Thus, liraglutide remains an effective though expensive treatment option in a country like Pakistan.
